# Lumbar Facet Joint Compressive Injury Induces Lasting Changes in Local Structure, Nociceptive Scores, and Inflammatory Mediators in a Novel Rat Model

**DOI:** 10.1155/2012/127636

**Published:** 2012-06-28

**Authors:** James L. Henry, Kiran Yashpal, Howard Vernon, Jaesung Kim, Hee-Jeong Im

**Affiliations:** ^1^Department of Psychiatry and Behavioural Neurosciences, McMaster University, HSC 4N35, 1200 Main Street West, Hamilton, ON, Canada L8N 3Z5; ^2^Division of Research, Canadian Memorial Chiropractic College, 6100 Leslie Street, Toronto, ON, Canada M2H 3J1; ^3^Department of Biochemistry, Rush University Medical Center, Cohn Research BD 516, 1735 W. Harrison, Chicago, IL 60612, USA; ^4^Section of Rheumatology, Department of Internal Medicine, Rush University Medical Center, Cohn Research BD 516, 1735 W. Harrison, Chicago, IL 60612, USA; ^5^Department of Orthopedic Surgery, Rush University Medical Center, Cohn Research BD 516, 1735 W. Harrison, Chicago, IL 60612, USA

## Abstract

*Objective*. To develop a novel animal model of persisting lumbar facet joint pain. *Methods*. Sprague Dawley rats were anaesthetized and the right lumbar (L5/L6) facet joint was exposed and compressed to ~1 mm with modified clamps applied for three minutes; sham-operated and naïve animals were used as control groups. After five days, animals were tested for hind-paw sensitivity using von Frey filaments and axial deep tissue sensitivity by algometer on assigned days up to 28 days. Animals were sacrificed at selected times for histological and biochemical analysis. *Results*. Histological sections revealed site-specific loss of cartilage in model animals only. Tactile hypersensitivity was observed for the ipsi- and contralateral paws lasting 28 days. The threshold at which deep tissue pressure just elicited vocalization was obtained at three lumbar levels; sensitivity at L1 > L3/4 > L6. Biochemical analyses revealed increases in proinflammatory cytokines, especially TNF-**α**, IL-1**α**, and IL-1**β**. *Conclusions*. These data suggest that compression of a facet joint induces a novel model of local cartilage loss accompanied by increased sensitivity to mechanical stimuli and by increases in inflammatory mediators. This new model may be useful for studies on mechanisms and treatment of lumbar facet joint pain and osteoarthritis.

## 1. Introduction

Low back pain is ubiquitous in Western society [1–3]. Its lifetime prevalence is generally accepted to be around 80% and is estimated to be one of the most costly of all medical conditions [1, 4, 5]. The majority of low back pain cases are considered to be nonspecific, with a mechanical origin [2, 6–8]. One of the structures of the spinal motion segment that has been implicated in mechanical low back pain is the lumbar facet joint; however, the contribution of facet joints to low back pain is still controversial [9–11]. Facet joints participate in load bearing in the lumbar spine during spinal motions and compressions [12–16]; they are well-innervated with nociceptors [17–26]; low back pain can be provoked in experimental conditions by irritation of the lumbar facet joints [27–29]. Anesthetic blockade can identify a contribution from facet joints in 15–67% of back pain cases [9, 11, 30–35]; neurotomy procedures can relieve chronic facet joint pain [36–39].

The human clinical studies cited above have major limitations with respect to investigating the underlying mechanisms of low back pain. While numerous animal models exist to investigate major spinal disorders [40–45], few animal models of lumbar facet joint injury exist [46–51]. To address the significant gaps in knowledge, we undertook to develop a rat model of mechanically induced lumbar facet joint injury. 

## 2. Materials and Methods

All experimental procedures were reviewed and approved by the McMaster University Animal Review Ethics Board, and animals were cared for and used according to the Guide to the Care and Use of laboratory Animals of the Canadian Council on Animal Care, Volumes 1 and 2.

### 2.1. Model Induction

Male Sprague Dawley rats (225–250, from Charles River Inc., St Constant, QC, Canada) were anesthetized with a combination of ketamine (5 mg/100 g), xylazine (0.5 mg/100 g), and aceptomazine (0.1 mg/100 g), i.p. A midline incision was made and the fascia along the right side of the supraspinous ligament was scraped by blunt dissection. The multifidus muscle at the L5 spinous process was similarly resected to expose the L5-L6 facet joint capsule. Subsequently the L5/6 facet joint was exposed unilaterally on the right side. In model animals (*n* = 6), the joint was compressed to ~1 mm with modified clamps applied for three minutes (average force = 400 grams); sham-surgery animals (*n* = 6) underwent exposure of the joint without application of the clamp. The muscle was then sutured, and the skin closed using suture clips. Antibiotic ointment (Nitrofurazone 0.2%) was applied over the wound, and 0.03 ml of the antibiotic Tribrissen 24% (trimethoprim-sulfadiazine) was injected subcutaneously. Animals were placed under a heating lamp until they recovered from the anaesthetic and then returned to their home cages. Following surgery, animals were allowed to recover and then were tested on days 5, 7, 14, 21, and 28 after surgery as described below. A third group of naïve control animals was also studied (*n* = 6). Baseline readings were taken before the induction of the model for mechanical sensitivity (von Frey Hair test) and pressure sensitivity (algometry).

### 2.2. Hind Paw Tactile Sensitivity Measured by Von Frey Filaments

Von Frey test of plantar sensitivity was included in this study in view of the radiation of pain sensitivity below the knee in some patients with low back pain [52], even to the foot [53]. Further, inflammation in the area of the vertebrae induces increased sensitivity of the foot in animals [54]. Testing consisted of applying an ascending series of fine, calibrated von Frey filaments to the plantar surface of a paw until a fibre is found from which a withdrawal response is observed. The method described previously in Pitcher et al. [55] was used. The total testing time for each rat usually lasted 35 to 40 min. Withdrawal thresholds were obtained before surgery and on days 5, 7, 14, 21, and 28 after model induction.

### 2.3. Pressure Sensitivity of Axial Tissues Measured by Algometer

For pressure algometry, the rat was acclimatized to a soft cloth and laid on a hard surface with a cylinder under its stomach so the back was slightly lifted. A pressure algometer [56] was then applied over the L6 spinous process and gently pressed until the animal demonstrated discomfort or vocalization. This process was repeated over the L3/4 and L1 spinous processes [23]. These three readings were taken again at 30 and 60 minutes later; this protocol was followed to avoid any additive effect of applying pressure. The data were averaged for each spinal level. Algometer data were obtained before surgery and on days 7, 14, 21, and 28 after model induction. The controls consisted of naïve (no surgery) and sham surgery groups.

### 2.4. Histology

Animals in each group were sacrificed for histological examination of lumbar vertebral segments on day 28 after all sensory testing had been completed (*n* = 3 per group).

For histological assessment, the tissue was fixed in 4% formaldehyde, followed by decalcification in EDTA, which was changed every 3 days for 6 weeks. The decalcified facet joints were then cut in the transverse plane, and paraffin-embedded. Serial facet joint sections of exact 5 *μ*m thickness were obtained and mounted on histological slides. Sections were taken through xylene and a descending series of alcohol baths to remove the paraffin ready for staining with Safranin-O Fast Green to assess general morphology and the loss of proteoglycan in cartilage ground substance. 

The staining solution, 0.1% (w/v) Safranin-O, was prepared in 0.1 M sodium acetate buffer at pH 4.6. Staining was carried out for 10 min, and the sections were dehydrated in ethanol solutions and cleared in xylene**. **Samples were then evaluated for cartilage status, joint surface smoothness, and joint space dimension at day 28. Since histological samples were obtained only on day 28, we graded the cartilage as “healthy” or “completely degenerated.”

### 2.5. Cytokine Antibody Array and Quantification

Selected animals were sacrificed for biochemical and molecular biology evaluation on days 7, 14, and 28 after model induction. The anesthetized rat was decapitated and the entire rat spinal cord ejected by pressure injection of physiological saline through the L6/S1 vertebral junction. The spinal dorsal horn was dissected under light microscope. The ipsilateral lumbar spinal dorsal horn was immediately wrapped into a piece of chilled-labeled foil and immersed into dry ice and kept frozen until use. These tissues were then subjected to either tissue extraction or total RNA extraction for cytokine antibody array, Western blotting, and real-time two PCR experiments, respectively. Intact naïve rats (*N* = 3) were included as a control.

Tissues were lysed by homogenization in RIPA buffer (150 mM NaCl, 1% NP-40, 0.5% deoxycholate, 0.1% SDS and 50 mM Tris, pH 7.5) with protease cocktail inhibitors (Sigma, St. Louis, MO, USA). The total protein concentrations of cell lysates were determined by a bicinchoninic acid protein assay (Pierce, Rockford, IL, USA). An array for cytokine proteins (Cytokine Array, RayBio, Norcross, GA, USA) was used to determine relative alterations in the level of cytokines. Membranes with immobilized antibodies were incubated for 14 h with either 500 *μ*g total protein of the sham control (asymptomatic) or experimental spinal tissues (symptomatic) extracted on day 7 or day 28, followed by biotin-conjugated antibodies, and further incubated with horseradish-peroxidase- (HRP-) conjugated streptavidin. Immunoreactivity was visualized using the ECL system (Amersham Biosciences, Piscataway, NJ, USA) and the Signal Visual Enhancer system (Pierce, Rockford, IL, USA), which magnifies the intensity of the signal. Densitometric measurement was performed by calculating the integrated density values for each spot (area relative intensity) by using Molecular Imager Versadoc MP 4000 System (Bio-Rad, Hercules CA, USA) and Quantity One-4.5.0 Basic 1-D Analysis Software (Bio-Rad, Hercules CA, USA). The positive control signals on each membrane were used in normalization of signal intensity.

### 2.6. Total RNA Isolation and Reverse Transcription and Real-Time PCR

Lumbar spinal dorsal horns were disrupted and homogenized. Total RNA was isolated from lumbar dorsal horns using the Trizol reagent (Invitrogen, Carlsbad, CA, USA) following the instructions provided by the manufacturer. 

Reverse transcription (RT) was carried out with 1ìg total RNA using ThermoScript TM RT-PCR system (Invitrogen, Carlsbad, CA, USA) for first strand cDNA synthesis. For real-time PCR, cDNA was amplified using MyiQ Real-Time PCR Detection System (Bio-Rad, Hercules CA, USA). Relative mRNA expression was determined using the 2[Δ][Δ]CT method, as detailed by manufacturer (Bio-Rad, Hercules CA, USA). Beta-actin was used as internal control. The primer sequences and the optimized conditions for use are summarized in Table 1.

### 2.7. Western Blot Analysis for ERK MAP Kinase Activity

Tissue lysates from the ipsi- and contralateral dorsal horns of the spinal cords were prepared using homogenizer and modified cell lysis RIPA buffer: 20 mM Tris (pH 7.5), 150 mM NaCl, 1 mM EDTA, 1 mM EGTA, 1% Nonidet P-40, 0.25% deoxycholate, 2.5 mM sodium pyrophosphate, 1 mM glycerol phosphate, 1 mM NaVO_4_, with 2 mM phenylmethylsulfonyl fluoride (Sigma, St. Louis, MO, USA). Total protein concentrations of spinal cord tissue lysates were determined by a bicinchoninic acid (BCA) protein assay (Pierce). Equal amount of protein was resolved by 10% SDS-PAGE gels and was transferred to nitrocellulose membrane for immunoblot analysis by using phosphospecific anti-ERK antibody (Cell Signaling, Danvers, MA). Nonphosphospecific total anti-ERK antibody (Cell Signaling, Danvers, MA) was used for internal control for normalization of the western blotting analyses. Immunoreactivity was visualized using the ECL system (Amersham) and the Signal Visual Enhancer system (Pierce) which magnifies the signal. All immunoblotting experiments were repeated at least three times. 

### 2.8. Statistical Analysis

All results are expressed as mean ± SEM. (standard error of mean). Statistical analysis on VF and algometry data was carried out using two-way repeated measures nested ANOVA's with the factors group and time. Post-hoc comparisons were conducted by evaluation of adjusted *P*-values using Tukey's test or Holm's method, depending on whether the analysis was pair-wise. *P* < 0.05 was accepted as significant in the ANOVA's. The evaluation of real-time PCR data was done by one-way ANOVA with a post-hoc Tukey's test using 2^∧^[∆][∆] Ct values of each sample. A value of *P* < 0.05 was considered significant.

## 3. Results

### 3.1. Histology of Lumbar Facet Joint

**Figure 1:**
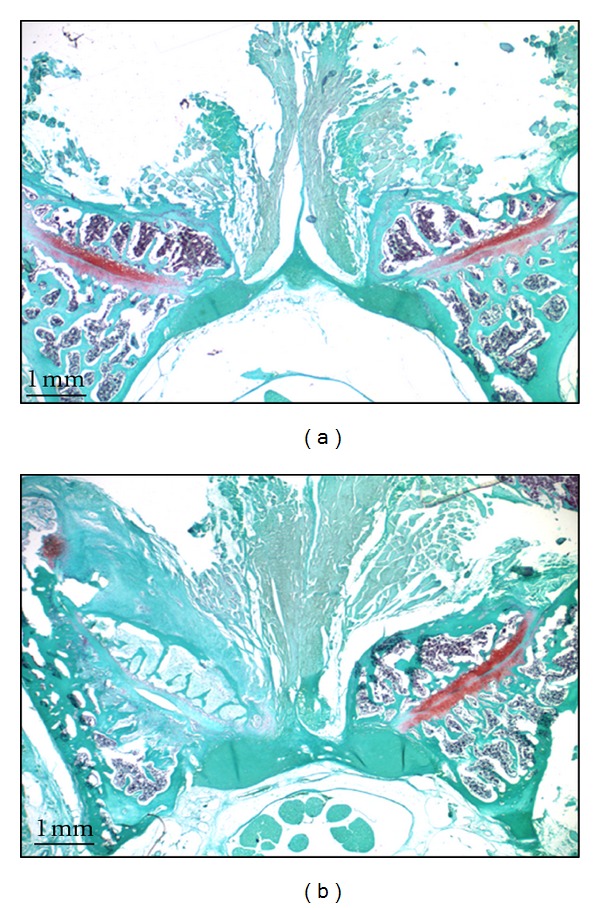
An axial section of the facet joint from L5/L6 stained with Safranin-O (4  wk post-surgery). (a)  * Left side: *sham control facet joint with exposed after open surgery, and *right side:* intact side of facet joint. (b) *Left side: *facet joint L5/L6 with open surgery followed by compression, and *right side:* intact side of facet joint (20x).

**Figure 2:**
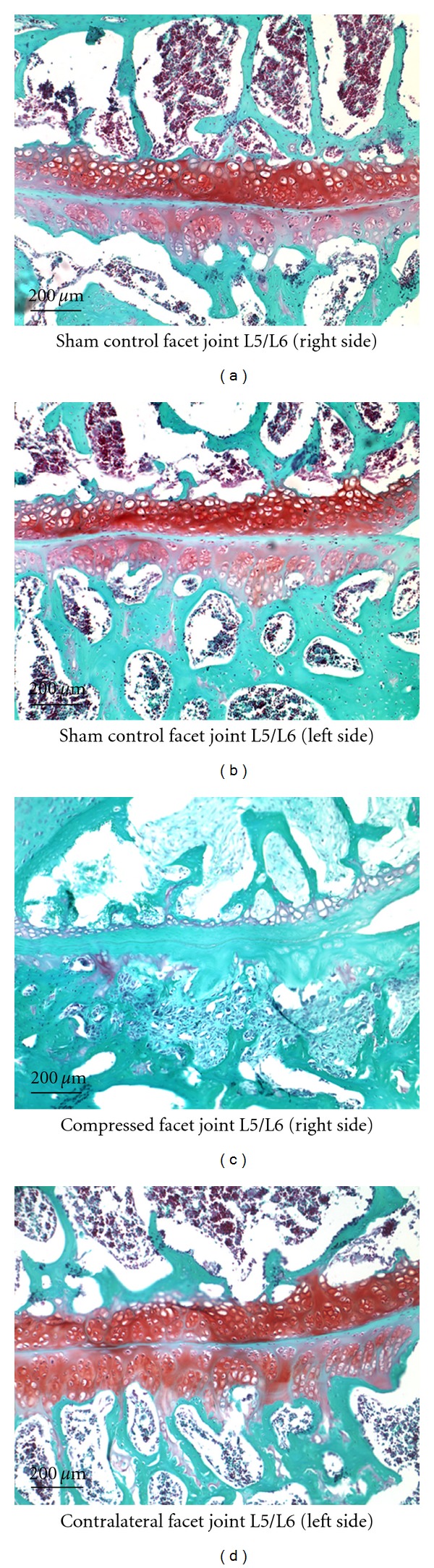
A larger magnification (100x) to examine structural changes in the facet joint L5/L6. Facet joints with and without compression were stained with Safranin-O (4 wk postsurgery) followed by microscopic examination.

In the model group, all samples scored “completely degenerated” while the sham surgery group samples all scored “healthy” cartilage. Histological examination of articular cartilage in the facet joint on both sides of sham control (left and right) Figures 1(a) and 1(b) and the contralateral joint in the model group (left) Figures 2(a)–2(d), shows no sign of tissue degeneration at any time. The articular surfaces were smooth and the matrix was densely stained (red) with Safranin-O. In the model group/compressed side, all rats demonstrated severe cartilage degeneration, proteoglycan loss, and structural changes in the ipsilateral facet joint components as reflected by surface irregularities and denudation at week 4.

### 3.2. Von Frey Test

**Figure 3:**
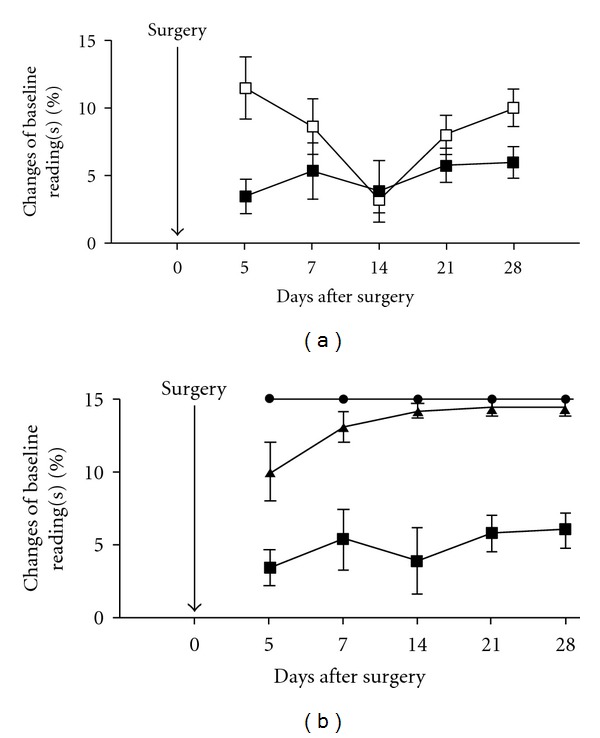
(a) von Frey data comparing tactile threshold between ipsilateral foot (■) with the contralateral foot (□) in animals after facet joint compression on day 5, 7, 14, 21, and 28. (b) Comparison of tactile sensitivity in the three groups: naïve (•; *n* = 6), sham (▲; *n* = 6) and model (■; *n* = 6) animals. The model animals showed a statistically significant difference from the sham and the naïve animals on all test days (*P* < 0.001).

The animals in the model group (*n* = 6) showed increased tactile sensitivity not only in the ipsilateral foot but also in the contralateral foot as shown in Figure 3(a). In the ipsilateral foot, the withdrawal threshold to von Frey filaments had decreased by day 5, and it remained lowered throughout the testing period, as measured on days 7, 14, 21, and 28. The data are shown as mean (±SEM) for each point. The withdrawal threshold of the contralateral foot also decreased, reaching its lowest level on day 14, after which it showed an upward return toward baseline by day 28.

Figure 3(b) illustrates the tactile sensitivity of the ipsilateral foot in the three animal groups: model (*n* = 6), sham surgery (*n* = 6) and naïve group with no surgery (*n* = 6). The naïve and sham groups remained at normal values on all days with no significant difference. As the figure shows, the model group is significantly different from both naïve and sham surgery groups on all the testing days (*P* < 0.0001). Post-hoc testing revealed that, at all time points, the model group's difference from the sham surgery and naïve groups was statistically significant (*P* values ranged from 0.0096–0.0001).

### 3.3. Algometer Test

**Figure 4:**
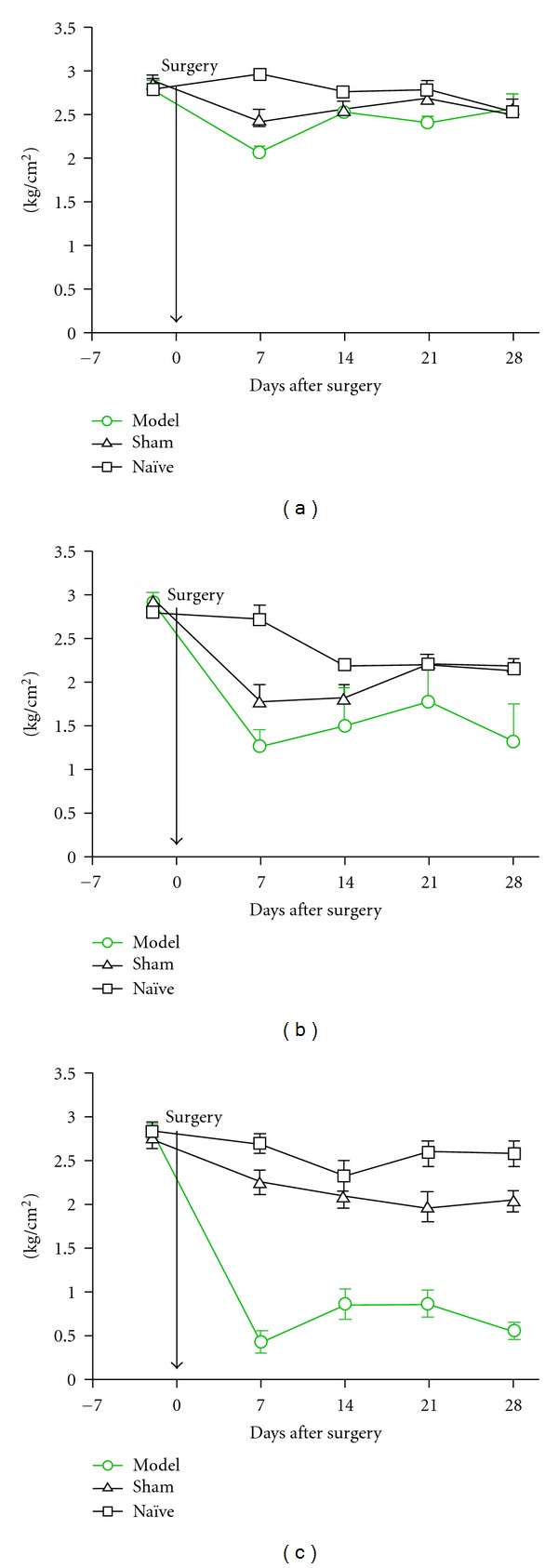
Pressure sensitivity measured by algometer in naive (*n* = 6), sham (*n* = 6) and model (*n* = 6) rats at lumbar levels 6, 3/4 and 1. (a) At L6, model animals showed a difference in sensitivity only on day 7 compared to either sham or naïve animals (*P* < 0.05). (b) At L3/4, model animals showed a higher sensitivity compared to sham animals on days 7 and 28 (*P* < 0.01) and compared to naïve animals on days 7, 14 and 28 (*P* < 0.01). (c) At L1, model animals showed a higher sensitivity to pressure on all test days compared to sham and naïve animals (*P* < 0.001).

The pressure sensitivity measured by algometer at L1, L3/4, and L6 in model rats (*n* = 6), sham rats (*n* = 6), and naïve rats (*n* = 6) is shown in Figure 4. The data are mean (SEM) for each lumbar position in each group. L6 appears to be the least sensitive site as shown in Figure 4(a). At L3/4, the model animals showed a significantly increased sensitivity compared to sham animals, but only on days 7 and 28 (*P* < 0.01). However, compared to naïve animals, the model animals were significantly more sensitive to pressure throughout the testing period (*P* < 0.01), except for day 21. The data are shown in Figure 4(b). At L1 model animals exhibited increased sensitivity compared to sham and naïve animals (*P* < 0.001) throughout the testing period, including days 7, 14, 21, and 28 (Figure 4(c)).

### 3.4. Cytokine Antibody Array

**Figure 5:**
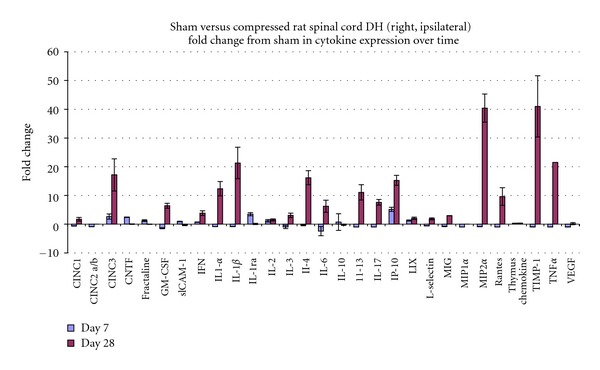
Cytokine array (all *P* values less than 0.05).

In the model group, prominent increases in the levels of multiple proinflammatory cytokines and chemokines were observed at day 28 (Figure 5;  all results *P* > 0.05). These cytokines include cytokine-induced neutrophil chemoattractant-3 (CINC3), IL-1*α*, IL-1*β*, RANTES, IL-6, IL-17, macrophage inflammatory protein 2*α* (MIP2*α*), and TNF*α*. Also, we observed significant induction of anti-inflammatory cytokines (e.g., IL-3, IL-4, IL-13) at day 28, but not at day 7. IL-1 receptor antagonist (IL-1ra) which can antagonize inflammatory action mediated by IL-1 cytokine family members is highly expressed at day 7 after the facet joint injury and returned to the control level at day 28. Unexpectedly, we observed a robust and sustained induction of tissue inhibitor of metalloproteinase-1 (TIMP-1), a potent inhibitor of matrix metalloproteinases (MMPs), at both Day 7 and Day 28 time points.

### 3.5. Real-Time PCR Analyses

**Figure 6:**
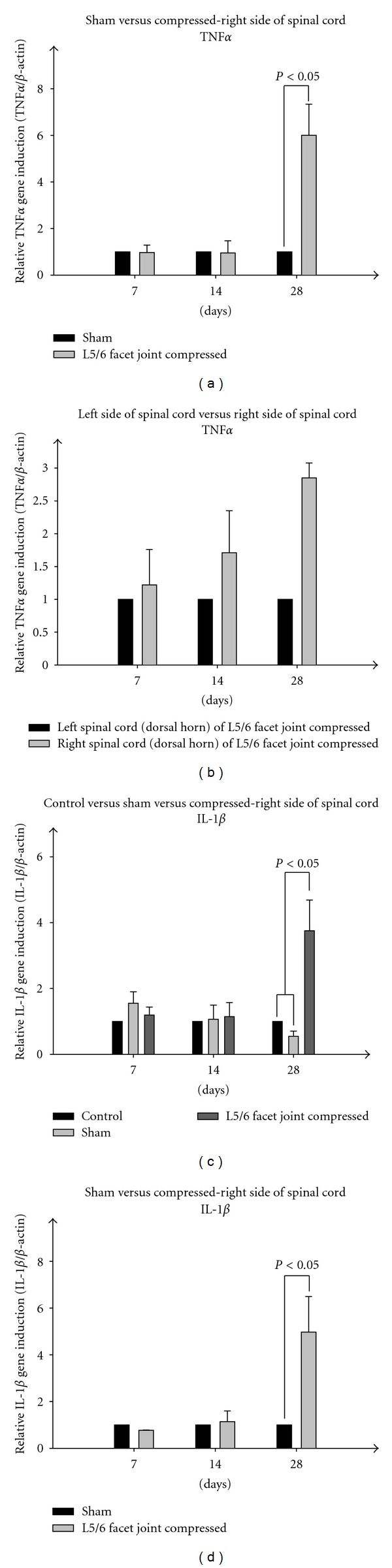
(a) Total dorsal horn TNF-alpha RNA. Sham control (gray, open surgery is performed as in the experimental group, but without facet joint compression) and the experimental group (black, open surgery followed by facet joint L5/L6 compression) during a time course (day 7, 14, and 28). Real-time PCR results were normalized by using *β*-actin as an internal control. (b) Total dorsal horn TNF-alpha RNA. Experimental group in a time course (day 7, 14, and 28), left versus right sides of lumbar spinal dorsal horn. Real-time PCR results were normalized by using *β*-actin as an internal control. (c) Total dorsal horn IL-1beta RNA. Sham control (gray, open surgery is performed as in the experimental group, but without facet joint compression) and the experimental group (black, open surgery followed by facet joint L5/L6 compression) during a time course (day 7, 14, and 28). Real-time PCR results were normalized by using *β*-actin as an internal control. (d)Total dorsal horn IL-1beta RNA. Experimental group in a time course (day 7, 14, and 28), left versus right sides of lumbar spinal dorsal horn. Real-time PCR results were normalized by using *β*-actin as an internal control.

We further assessed whether cytokine protein levels correspond with changes in mRNA levels within the cellular components of the spinal cord (i.e. glial cells and neurons). We examined IL-1beta and TNF*α* mRNA as representative pain-associated cytokines which are highly upregulated at the protein level in the spinal cord due to facet joint compression-induced pain (Figures 6(a)–6(d)). Real-time PCR results demonstrate that the mRNA level of TNF*α* is substantially increased at the chronic stages of facet joint injury-induced pain period (*P* < 0.05, Day 28), but not during earlier stages (day 7 or day 14). As expected, highly upregulated expression of TNF*α* was observed at the right spinal cord dorsal horn of compressed facet joint, (*P* < 0.05, day 28) compared to left spinal cord dorsal horn at the same level.

We observed almost identical expression patterns for IL-1*β* in the spinal cord. Significantly induced expression of IL-1*β* at day 28 time point after facet joint injury (*P* < 0.05) was detected which is not observed during earlier time points (day 7 or day 14). We also observed highly upregulated expression of IL-1*β* at the right spinal cord dorsal horn of compressed facet joints (*P* < 0.05, day 28) compared to left spinal cord dorsal horn at the model level. Parallel experiments were performed using spinal samples from controls (surgery and naïve) in which we found no significant differences in the mRNA levels of either TNF*α* or IL-1*β* throughout the experimental time course. Notably, these mRNA expression levels are consistent with protein levels detected in cytokine antibody array results (Figure 5).

### 3.6. ERK MAP Kinase Activity in Dorsal Horn of the Spinal Cords

**Figure 7:**
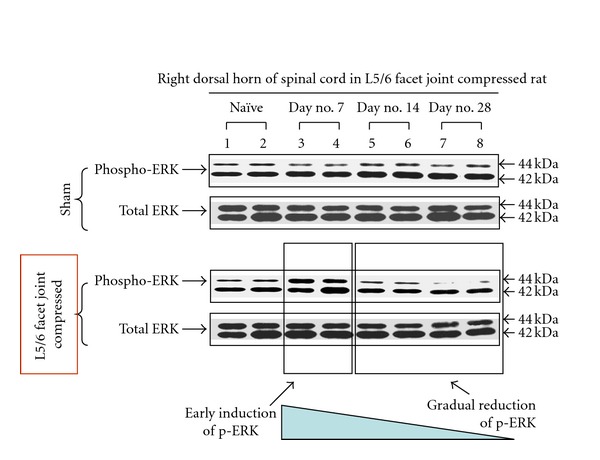
Entire rat spinal cords were ejected and lumbar dorsal horns were dissected from intact control (naïve tissue), sham control (upper panel, open surgery is performed as in the experimental group, but without facet joint compression) and the experimental group (lower panel, open surgery followed by facet joint L5/L6 compression) in a time course (day 7, 14, and 28). Tissue lysates of spinal cords were prepared and equal amount of protein (25 *μ*g each) was analyzed for the activation of ERK MAP kinase 1/2 (44 and 42 kDa, resp.) by using phosphospecific anti-ERK1/2 antibody. Nonphosphospecific total anti-ERK antibody was used for internal control for normalization of the western blotting analyses. All immunoblotting experiments were repeated at least three times.

ERK/MAPK levels in the lumbar spinal dorsal horn (sham control (upper panel) versus experimental group (lower panel): days 7, 14, and 28) are shown in Figure 7. Compared to naïve controls (*lanes 1, 2*) and sham-surgery controls (upper panel, *lanes 3, 4*), we observed that the early induction of ERK activation within day 7 reflected by phosphorylation of a 44 kDa MAPK isoform (ERK1/MAPK) in the experimental group (L5/L6 facet joint compressed) (lower panel, *lanes 3, 4*). These early inductions of ERK are significantly decreased in a time-dependent manner (lower panel, *lanes 5–8*) whereas no change is observed in naïve controls.

## 4. Discussion

The present study demonstrates that a brief compression of the right L5-L6 facet joint in the rat produces a localized intra-articular damage as evidenced by the substantial degradative changes in facet joint cartilage by day 28 as compared to sham surgery and naïve control animals. The histological findings of severe cartilage degeneration at 28 days compare favorably to those of Tachihara et al. [46], who used injection of Complete Freund's Adjuvant, Kim et al. [51]—who used monosodium iodoacetate (MIA) and to those of Yeh et al. [57], who injected collagenase into a single lumbar facet joint. 

Our model also exhibited pain-related behavioral changes. For this analysis, we employed one standard test—von Frey hairs for tactile hypersensitivity—as well as one novel test—pressure algometry over the spine. Our finding of sustained ipsilateral hypersensitivity, lasting at least to 28 days, is in contrast to that of Tachihara et al. [46] who reported model versus control differences only up to 7 days. The first week postsurgery and postmodel induction could be considered as a time when such behavioral signs represent reactions primarily to these interventions. It is of great importance, then, that the hypersensitivity induced by mechanical compression of the facet joint in the current model does persist beyond this stage and likely represents a manifestation of sustained nociceptive input from the injured facet joint rather than possible postsurgical input.

These findings are consistent with those of Lee et al. [58–61] who have demonstrated similar findings of tactile hypersensitivity in the forepaw of a rat model of cervical facet joint mechanical injury up to 14 days postmodel induction.

Our finding of bilateral tactile hypersensitivity is novel, and suggests substantial spill-over of nociceptive input to the contralateral dorsal horn [62, 63]. Bilateral hypersensitivity argues against the explanation in Tachihara et al. [46] that their finding of unilateral hypersensitivity was the result of inflammatory exudation anterior to the facet joint irritating the nerve root, thus inducing a radiculopathy.

The algometry data are, to our knowledge, relatively novel within the group of studies of facet joint models. Only Kim et al. [51] have reported using spinal algometry; however, they measured pressure pain threshold at only the lesion site. In the present study, for all test sites, model rats demonstrated significantly greater reductions in local spinal pressure thresholds for all test points, indicating, once again, that mechanical facet compression produces long-lasting changes that would be equivalent to low back “tenderness” in the human circumstance. It is noteworthy that the spinal pressure hypersensitivity also exhibits a spatial spread, whereby the greatest reduction in pressure threshold in model animals was at the L1-L2 vertebral level. This finding could be explained by the fact that the L5-6 facet joint (at least in the rat) has been shown to receive innervation from L2 via afferents which descend from that level in the paravertebral sympathetic trunk [23, 64]. This has been used to explain referred pain to the groin in cases of L5-6 disc and facet pain [23]. Alternatively, the application of the pressure algometer at the L1-2 level may have resulted in larger bending moments at the L5-6 level with greater irritation of the model lesion.

The biochemical findings suggest that modulation in the expression of proinflammatory cytokines, such as IL-1*β* or TNF*α*, and perhaps combinations of other cytokines, in dorsal horn neurons, is induced by mechanical facet joint compression and persists up to 28 days. Our findings cannot be directly compared to those of Tachihara et al. [46] because they only measured TNF*α* expression in dorsal root gangion cells, while we studied dorsal horn cell expression. Interestingly, they found upregulation in TNF-alpha-expressing DRG neurons for only days 1 and 3; no difference was found between model versus controls from days 7–28. In our study, TNF-alpha and IL-1-*β* expression was largest in DH cells at day 28. Our findings are consistent with those of Lee et al. [65, 66] who demonstrated increased cytokine mRNA levels in the spinal cord of their cervical facet-injured rats. 

Spinal cord ERK responses are a novel marker for facet pain studies. Mitogen-activated protein kinases (MAPKs), which encompass the three subgroups, ERK, p38, and JNK MAPKs, are important for intracellular signal transduction and play critical roles in regulating neural plasticity and inflammatory responses. In particular, ERK activation in spinal cord dorsal horn neurons by nociceptive activity plays a critical role in central sensitization by regulating the activity of glutamate receptors and potassium channels [67–73].

To our knowledge, this is the first animal model of mechanically induced facet joint pain to demonstrate cartilage degeneration. This is in contrast to prior models which induced an autoimmune reaction in the joint (with CFA [46]), cartilage cell apoptosis with collagenase injection [57], chondrocyte disruption with MIA [51], or pain with surgical incision [49, 50]. In the case of CFA injection, while a more rapid onset of autoimmune-induced inflammatory reaction appears to be induced, with signs of cartilage degeneration appearing within 3 days, significant differences in nociceptive behaviors in model animals are demonstrated for only 7 days postmodel induction. In the work of Yeh et al. [57], findings of cartilage degeneration are evident by day 7; however, no data were presented on nociceptive behaviors. In the works of Miyagi et al. [49] and Sakuma et al. [50], only nociceptive-related findings were reported with no indications of facet joint arthritic changes. Kim et al. [51] report on nociceptive-related and histological changes but do not report on dorsal horn biomarkers. Our model appears to create significant changes in each of these domains, pain behaviors and mechanisms as well as degenerative joint changes, and it appears to do so in a timeframe that more closely emulates some conditions of low back pain in humans.

To provide more details on the time course of the end-points in this study and to examine the correlation between the histological, biochemical, and behavioral changes, we report here, future studies could aim at larger sample sizes as well as additional time points.

## 5. Conclusions

The results of this initial study are encouraging and prompt further study on this model. A brief physical trauma to a single facet joint induces important structural and functional changes. The initial biochemical studies confirm that changes occur in mechanisms related to adaptive and maladaptive reactions to brief trauma. The results of this study may provide a basis for future investigation, understanding, and, eventually, treatment of lumbar facet-related pain.

## Figures and Tables

**Table 1:** 

Primer	Sequences	Tm	Gene No.
TNF-*α*	Forward: 5^′^-TCTGTGCCTCAGCCTCTTCTCATT-3^′^	60	NM_012675.3
	Reverse: 5^′^-TTGGGAACTTCTCCTCCTTGTTGG-3^′^		
IL-1*β*	Forward: 5^′^-TCATCTTTGAAGAAGAGCCCGTCC-3^′^	60	NM_031512.2
	Reverse: 5^′^-TGCAGTGCAGCTGTCTAATGGGAA-3^′^		
CGRP	Forward: 5^′^-TCTAGTGTCACTGCCCAGAAGAGA-3^′^	55	NM_001033956.1
	Reverse: 5^′^-GGCACAAAGTTGTCCTTCACCACA-3^′^		
Substance P	Forward: 5^′^-TGGTCAGATCTCTCACAAAGG-3^′^	55	NM_012666.2
	Reverse: 5^′^-TGCATTGCGCTTCTTTCATA-3^′^		
MMP-2	Forward: 5^′^-ACCTCTTACAACAGCTGTACCACC-3′	60	NM_031054.2
	Reverse: 5^′^-TTTCCACCCACAGTGGACATAGCA-3′		
BDNF	Forward: 5^′^-TCCTGGAGAAAGTCCCGGTATCAA-3^′^	60	GQ395803.1
	Reverse: 5′-TAGTTCGGCATTGCGAGTTCCAGT-3^′^		
NK-1	Forward: 5^′^-TGGGCAACGTAGTGGTGATA-3^′^	60	NM_012667.2
	Reverse: 5^′^-CACGGCTGTCATGGAGTAGA-3^′^		
NK-2	Forward: 5^′^-CCGAGCACCATTCTGTTTTT-3^′^	60	NM_080768.1
	Reverse: 5^′^-GGAGAGTCAACCGGTGTCAT-3^′^		
Galanin	Forward: 5^′^-TTCCCACCACTGCTCAAGATG-3^′^	55	NM_033237.1
	Reverse: 5^′^-TGGCTGACAGGGTTGCAA-3^′^		
Neuropeptide Y	Forward: 5^′^-AGATCCAGCCCTGAGACACTGATT-3^′^	55	M15793.1
	Reverse: 5^′^-TGGAAGGGTCTTCAAGCCTTGTTC-3^′^		
*β*-actin	Forward: 5^′^-TGTCACCAACTGGGACGATATGGA-3^′^	55	NM_031144
	Reverse: 5^′^-AGCACAGGGTGCTCCTCA-3^′^		
